# Association of antioxidants use with the risk of dementia among community-dwelling adults in the United Kingdom biobank

**DOI:** 10.3389/fnut.2023.1270179

**Published:** 2024-01-04

**Authors:** Xianwen Shang, Jiahao Liu, Xueli Zhang, Yu Huang, Zhuoting Zhu, Shulin Tang, Wei Wang, Zongyuan Ge, Honghua Yu, Mingguang He

**Affiliations:** ^1^Guangdong Eye Institute, Department of Ophthalmology, Guangdong Provincial People's Hospital (Guangdong Academy of Medical Sciences), Southern Medical University, Guangzhou, China; ^2^Center for Eye Research Australia, Royal Victorian Eye and Ear Hospital, Melbourne, VIC, Australia; ^3^State Key Laboratory of Ophthalmology, Zhongshan Ophthalmic Center, Sun Yat-sen University, Guangzhou, China; ^4^Monash e-Research Center, Faculty of Engineering, Airdoc Research, Nvidia AI Technology Research Center, Monash University, Melbourne, VIC, Australia; ^5^Experimental Ophthalmology, The Hong Kong Polytechnic University, Kowloon, Hong Kong SAR, China

**Keywords:** zinc supplement, vitamin E supplement, dementia, vitamin C supplement, moderation analysis

## Abstract

**Background:**

Data regarding the association between antioxidant supplementation and incident dementia are limited.

**Methods:**

We included 494,632 adults (54.5% females) aged 40–71 years at baseline from the United Kingdom Biobank in the final analysis. Incident dementia was ascertained using hospital inpatient and death records up to January 2021.

**Results:**

Over a median follow-up of 11.9 years, 7,128 new cases of all-cause dementia, 2,772 cases of Alzheimer’s disease, and 1,397 cases of vascular dementia were recorded. The hazard ratio (95% CI) for incident dementia associated with zinc supplementation was 0.84 (0.74–0.96), and the association remained significant after adjusting for all confounders (0.84 (0.74–0.96)). In the full model, zinc supplementation was associated with a reduced risk of Alzheimer’s disease [HR (95% CI): 0.71 (0.57–0.88)]. There was no significant association between zinc supplementation and the risk of vascular dementia. No significant associations with incident dementia were observed for other antioxidant supplementation. The association between zinc supplementation and incident dementia was significant among individuals with [HR (95% CI): 0.34 (0.15–0.77)] and without cataract [0.87 (0.77–0.99)] but it was stronger among those with cataract (*p* value for interaction = 0.0271).

**Conclusion:**

Our findings suggest that zinc supplementation may help reduce the risk of all-cause dementia and Alzheimer’s disease in middle-aged or older adults, especially among those with cataracts.

## Introduction

Free radicals, known as harmful compounds produced by natural biological processes in our body, play a critical role in the pathogenesis of chronic diseases including cardiovascular diseases, diabetes, and neurodegenerative diseases (1, 2). Maintaining a proper balance between oxidants and antioxidants may help protect against the harmful effects of these free radicals (1). Numerous studies have investigated the association between the antioxidant supplementation, which includes vitamins, selenium, and zinc, and the risk of cardiovascular disease, diabetes, and cancer, yielding inconsistent results (3, 4).

Antioxidant properties may help neutralize free radicals (1, 2), reduce inflammation (1), lower neuronal damage and functional deficits (1), thus mitigating the risk of neurological disorders. Antioxidants may enhance blood flow to the brain, which is crucial for delivering oxygen and nutrients to brain cells (5) and is associated with a lower risk of brain damage and cognitive impairment. While antioxidants may play an important role in the development of neurological disorders (1, 2, 6), it is unclear regarding the association between antioxidants intake and dementia. Previous studies have yielded inconsistent results regarding the association between vitamins supplementation and cognitive function (7). Several recent cross-sectional studies have linked zinc and selenium intake to cognitive function in older adults (8, 9). However, less is known regarding the association between antioxidants supplementation and the risk of dementia.

Using the United Kingdom Biobank, we aimed to examine the association of antioxidant supplementation with incident dementia. Additionally, we assessed whether this association varied across subgroups of important dementia risk factors.

## Methods

### Study population

The United Kingdom Biobank comprises a population-based cohort of over 500,000 individuals, all aged between 40 and 73 years at the baseline data collection conducted from 2006 to 2010 (10). Out of approximately 9.2 million eligible individuals aged 40–73 years, who were registered with the National Health Service, data from 502,505 individuals were collected at baseline. The participants attended one of the 22 assessment centers throughout the United Kingdom (10). Baseline data were linked to hospital inpatient and mortality register records using information including National Health Service number, surname, date of birth, sex, and address postcode.

The United Kingdom Biobank Study’s ethical approval has been granted by the National Information Governance Board for Health and Social Care and the NHS North West Multicenter Research Ethics Committee. All participants provided informed consent through electronic signature at recruitment.

### Supplementation of antioxidants

Supplement use of dietary antioxidants was defined based on a touchscreen question “Do you regularly take any of the following?” Participants selected more than one answer from a list of supplements. We defined antioxidants users by answering “yes” for the following dietary supplements: vitamin C, vitamin E, selenium, and zinc.

### Ascertainment of incident dementia

Dementia cases at baseline were identified through the examination of hospital inpatient records and self-reported information. To identify incident dementia cases, we analyzed hospital inpatient records and mortality register data. Cases of dementia, with primary or secondary diagnoses, were identified using the ICD coding system (detailed in Supplementary Table S1). Additional cases of dementia were determined by identifying cases where dementia was classified as the underlying or contributory cause of death. The onset date of dementia was established by using the earliest recorded date, irrespective of the data source. Person-years were calculated by measuring the time from the baseline assessment to the date of dementia onset, date of mortality, or the end of the follow-up period (England and Wales: 31 December 2020, Scotland: 31 January 2021), whichever came first.

### Covariates

All covariates included in the analysis were assessed at baseline. Demographic information (age, sex, ethnicity, education, and income), and lifestyle factors (sleep duration, physical activity, smoking, and diet) were collected using a touchscreen questionnaire. Excess metabolic equivalent (MET)-hours/week of physical activity during work and leisure time was assessed using the short form of the International Physical Activity Questionnaire (11). A healthy diet score was computed by evaluating seven commonly consumed food groups, following dietary guidelines aimed at promoting cardiometabolic health (12). A higher score indicated a healthier diet.

Chronic conditions at baseline were identified through self-reported data or participant interviews. Body mass index (BMI) was determined using measured weight and height, and obesity was defined as having a BMI ≥ 30 kg/m^2^ (13). The APOE E genotype was directly determined through genotyping, which involves analyzing two specific single nucleotide polymorphisms (rs7412 and rs429358). The presence of APOE4 was defined using the APOE4+ dominant model, which includes individuals with the E3/E4 and E4/E4 genotypes.

### Statistical analysis

Baseline characteristics data were presented as frequency (percentage) for categorical variables and means ± standard deviations for continuous variables by sex. To assess the differences in baseline characteristics between sexes, T-tests were conducted for continuous variables, and Chi-square tests were employed for categorical variables.

Cox proportional hazard regression models were utilized to explore the relationship between individual antioxidant supplementation and the occurrence of incident dementia and its various phenotypes. We tested three models: (1) age and sex; (2) Model 1 plus APOE4 status, ethnicity, education, income, diet, physical activity, smoking habits, alcohol consumption, sleep duration, BMI, depression, dyslipidemia, hypertension, diabetes, and stroke at baseline; (3) Model 2 plus calcium, glucosamine, and fish oil supplements (These supplements might be associated with the development of dementia and might be an indicator of biological aging status). We assumed that the association between antioxidant supplementation and incident dementia might differ between individuals at different risk levels of dementia. Therefore, we examined the relationship between antioxidants supplementation and incident dementia, stratified by dementia risk factors such as age, sex, APOE4 status, education, diet score, depression, dyslipidemia, hypertension, diabetes, and stroke.

A sensitivity analysis was conducted among zinc supplement users and age- and sex-matched non-users (1–3).

For categorical variables, missing values were consolidated into a single category. For continuous covariates, missing values were imputed with the mean.

Data analyses were conducted using SAS 9.4 for Windows (SAS Institute Inc.) and all *p* values were two-sided with statistical significance set at <0.05.

## Results

### Population selection and baseline characteristics

After excluding individuals who were lost to follow-up (*n* = 1,260), those with missing data on antioxidants (*n* = 6,195), or those with prevalent dementia (*n* = 412), the final analysis included 494,632 adults (54.5% females) aged 40–71 years (mean ± SD: 56.6 ± 8.1). In comparison to individuals who did not develop dementia during follow-up, those with incident dementia were more likely to have lower levels of education, be non-alcohol drinkers, be current smokers and be physical in activity. They also exhibited a higher prevalence of hypertension, diabetes, stroke, and dyslipidemia but a lower prevalence of depression (all *p* values<0.05, Table 1).

**Table 1:** Study population characteristics by incident dementia.

	Incident dementia		*p* value^*^
	No	Yes	
Age (years)	56.4 ± 8.1	63.8 ± 5.3	<0.0001
Sex			<0.0001
Women	266,043 (54.6)	3,300 (45.9)	
Men	221,398 (45.4)	3,891 (54.1)	
Ethnicity			<0.0001
Whites	459,797 (94.3)	6,870 (95.5)	
Non-whites	25,978 (5.3)	289 (4.0)	
Missing	1,666 (0.3)	32 (0.4)	
APOE4			<0.0001
No	363,340 (74.5)	4,042 (56.2)	
Yes	113,871 (23.4)	2,979 (41.4)	
Missing	10,230 (2.1)	170 (2.4)	
Education			<0.0001
0–5 years	82,067 (16.8)	2,488 (34.6)	
6–12 years	241,636 (49.6)	3,089 (43.0)	
≥13 years	158,710 (32.6)	1,461 (20.3)	
Missing	5,028 (1.0)	153 (2.1)	
Household income (pounds)			<0.0001
<18,000	93,873 (19.3)	2,550 (35.5)	
18,000–30,999	106,026 (21.8)	1,654 (23.0)	
31,000–51,999	109,501 (22.5)	830 (11.5)	
52,000–100,000	85,506 (17.5)	373 (5.2)	
>100,000	22,693 (4.7)	89 (1.2)	
Unknown	20,292 (4.2)	680 (9.5)	
Not answered	49,550 (10.2)	1,015 (14.1)	
Physical activity (MET-minutes/week)	2,655 ± 2,439	2,686 ± 2,551	0.28
Diet score	3.88 ± 1.44	3.79 ± 1.46	<0.0001
Sleep duration (hours)	7.15 ± 1.10	7.24 ± 1.36	<0.0001
Alcohol consumption			<0.0001
Never	21,389 (4.4)	477 (6.6)	
Previous	17,253 (3.5)	531 (7.4)	
Current	448,799 (92.1)	6,183 (86.0)	
Smoking			<0.0001
Never	268,548 (55.1)	3,296 (45.8)	
Former	167,784 (34.4)	3,034 (42.2)	
Current	51,109 (10.5)	861 (12.0)	
BMI (kg/m^2^)	27.42 ± 4.73	27.81 ± 4.88	<0.0001
Depression	27,501 (5.6)	653 (9.1)	<0.0001
Hypertension	128,026 (26.3)	3,192 (44.4)	<0.0001
Dyslipidemia	59,335 (12.2)	1,697 (23.6)	<0.0001
Diabetes	23,916 (4.9)	1,049 (14.6)	<0.0001
Stroke	8,232 (1.7)	474 (6.6)	<0.0001
Supplement			
Fish oil	152,788 (31.3)	2,591 (36.0)	<0.0001
Calcium	34,131 (7.0)	627 (8.7)	<0.0001
Glucosamine	92,804 (19.0)	1,484 (20.6)	0.0006

The data are presented as means ± standard deviations or frequency (percentage). APOE4, Apolipoprotein E Ɛ4; BMI, Body mass index; MET, Metabolic equivalent.

^*^The *t*-test was utilized to assess the difference of continuous variables between individuals with and without incident dementia, while the Chi-square test was employed for categorical variables.

### Incidence of dementia

Over a median follow-up of 11.9 years (interquartile range: 11.2–12.6), 7,128 cases of incident all-cause dementia, 2,772 cases of Alzheimer’s disease, and 1,397 cases of vascular dementia were documented.

### Antioxidants supplementation and incident dementia

**Figure 1:**
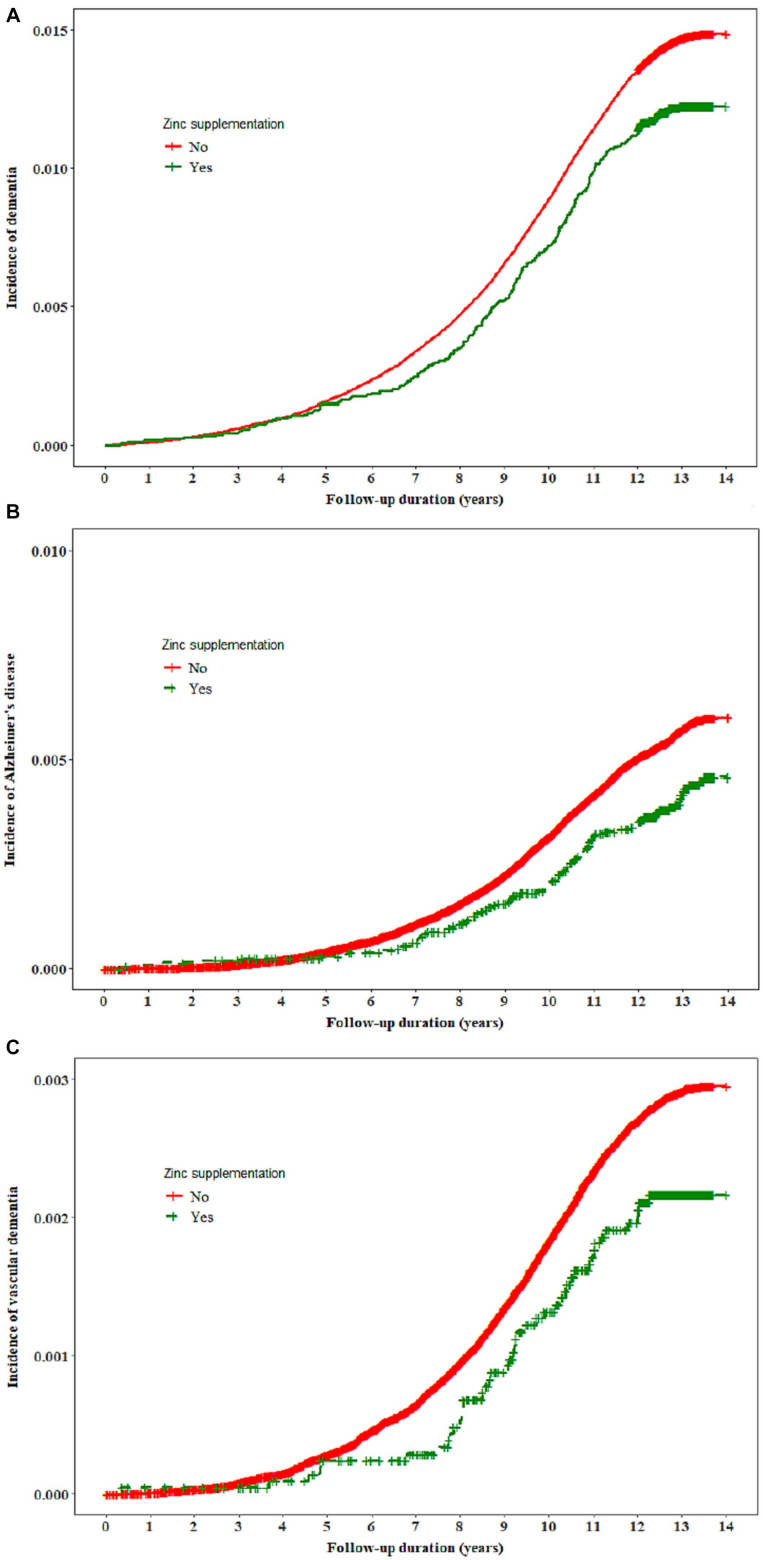
The incidence of all-cause dementia, Alzheimer’s disease, and vascular dementia by zinc supplementation.

There was no significant association between vitamin C, vitamin E, or selenium supplementation and the risk of dementia. Individuals who received zinc supplementation exhibited a lower incidence of dementia and Alzheimer’s disease compared to those who did not receive any supplementation (Figure 1). The hazard ratio ([HR] 95% confidence interval [CI]) for the risk of dementia associated with zinc supplementation was 0.84 (0.74–0.96) and this association remained significant when all confounders were adjusted for [0.84 (0.74–0.96)]. In the full model, zinc supplementation was associated with a lower risk of Alzheimer’s disease [HR (95% CI): 0.71 (0.57–0.88)]. We did not find a significant association between zinc supplementation and the incidence of vascular dementia (Table 2).

**Table 2:** Antioxidants supplementation and the risk of incident dementia.

	Antioxidant use		*p* value
	No	Yes	
**Dementia**			
Vitamin C			
Events	6,428	700	
Person-years	5,338,874	514,350	
HR (95% CI), Model 1	Reference	1.05 (0.97–1.14)	0.19
HR (95% CI), Model 2	Reference	1.07 (0.99–1.16)	0.0782
HR (95% CI), Model 3	Reference	1.08 (1.00–1.17)	0.0528
Vitamin E			
Events	6,874	254	
Person-years	5,674,882	178,342	
HR (95% CI), Model 1	Reference	1.05 (0.93–1.19)	0.44
HR (95% CI), Model 2	Reference	1.09 (0.96–1.23)	0.19
HR (95% CI), Model 3	Reference	1.09 (0.96–1.24)	0.17
Zinc			
Events	6,943	248	
Person-years	5,631,279	242,457	
HR (95% CI), Model 1	Reference	0.84 (0.74–0.96)	0.0087
HR (95% CI), Model 2	Reference	0.86 (0.76–0.97)	0.0181
HR (95% CI), Model 3	Reference	0.84 (0.74–0.96)	0.0087
Selenium			
Events	6,986	205	
Person-years	5,733,740	139,996	
HR (95% CI), Model 1	Reference	1.00 (0.87–1.15)	0.96
HR (95% CI), Model 2	Reference	1.09 (0.94–1.25)	0.25
HR (95% CI), Model 3	Reference	1.09 (0.95–1.26)	0.23
**Alzheimer’s disease**			
Vitamin C			
Events	2,497	275	
Person-years	5,338,874	514,350	
HR (95% CI), Model 1	Reference	1.05 (0.93–1.19)	0.44
HR (95% CI), Model 2	Reference	1.05 (0.93–1.19)	0.42
HR (95% CI), Model 3	Reference	1.04 (0.91–1.18)	0.60
Vitamin E			
Events	2,680	92	
Person-years	5,674,882	178,342	
HR (95% CI), Model 1	Reference	0.94 (0.76–1.16)	0.57
HR (95% CI), Model 2	Reference	0.96 (0.78–1.18)	0.69
HR (95% CI), Model 3	Reference	0.93 (0.75–1.15)	0.51
Zinc			
Events	2,710	87	
Person-years	5,631,279	242,457	
HR (95% CI), Model 1	Reference	0.75 (0.60–0.93)	0.0077
HR (95% CI), Model 2	Reference	0.75 (0.60–0.93)	0.0079
HR (95% CI), Model 3	Reference	0.71 (0.57–0.88)	0.0023
Selenium			
Events	2,717	80	
Person-years	5,733,740	139,996	
HR (95% CI), Model 1	Reference	0.99 (0.80–1.24)	0.96
HR (95% CI), Model 2	Reference	1.04 (0.84–1.31)	0.70
HR (95% CI), Model 3	Reference	1.01 (0.81–1.27)	0.91
**Vascular dementia**			
Vitamin C			
Events	1,274	123	
Person-years	5,338,874	514,350	
HR (95% CI), Model 1	Reference	0.93 (0.77–1.12)	0.42
HR (95% CI), Model 2	Reference	0.98 (0.82–1.18)	0.87
HR (95% CI), Model 3	Reference	1.01 (0.83–1.22)	0.93
Vitamin E			
Events	1,356	41	
Person-years	5,674,882	178,342	
HR (95% CI), Model 1	Reference	0.86 (0.63–1.18)	0.36
HR (95% CI), Model 2	Reference	0.94 (0.69–1.29)	0.72
HR (95% CI), Model 3	Reference	0.97 (0.70–1.33)	0.84
Zinc			
Events	1,371	44	
Person-years	5,631,279	242,457	
HR (95% CI), Model 1	Reference	0.77 (0.57–1.04)	0.0933
HR (95% CI), Model 2	Reference	0.82 (0.61–1.11)	0.21
HR (95% CI), Model 3	Reference	0.82 (0.60–1.12)	0.21
Selenium			
Events	1,384	31	
Person-years	5,733,740	139,996	
HR (95% CI), Model 1	Reference	0.76 (0.53–1.08)	0.13
HR (95% CI), Model 2	Reference	0.88 (0.62–1.26)	0.48
HR (95% CI), Model 3	Reference	0.91 (0.63–1.30)	0.60

We estimated the hazard ratio (95% CI) for incident dementia related to antioxidant supplementation using Cox proportional regression models. Model 1 was adjusted for age and sex; Model 2 was adjusted for Model 1 and APOE4 status, ethnicity, education, income, diet score, smoking, alcohol consumption, sleep duration, physical activity, BMI, depression, dyslipidemia, hypertension, diabetes, and stroke at baseline; Model 3 was adjusted for Model 2 and calcium, glucosamine, and fish oil supplements.

### Moderation analysis

**Figure 2:**
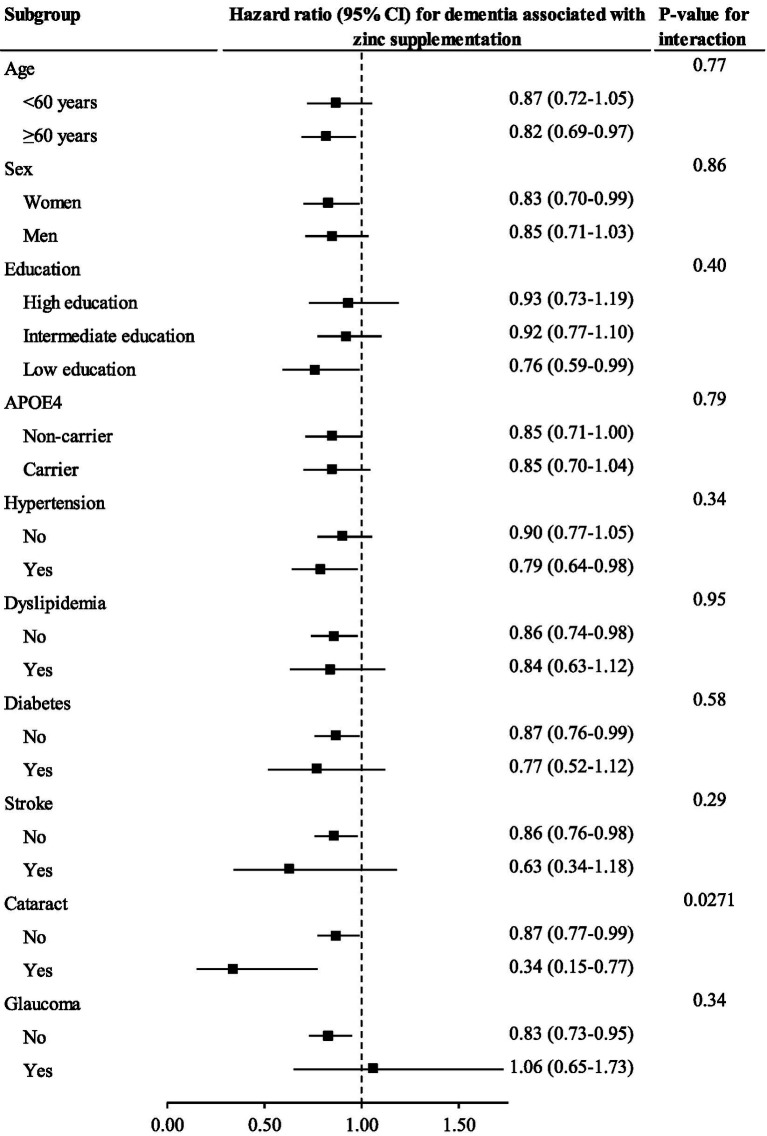
The association between zinc supplementation and incident dementia stratified by important factors Cox regression models were used to estimate the hazard ratio (95% CI) for incident dementia associated with zinc supplementation stratified by important factors.

The association between zinc supplementation and incident dementia was significant among individuals with [HR (95% CI): 0.34 (0.15–0.77)] and without cataract [0.87 (0.77–0.99)] but it was stronger among those with cataract (*p* value for interaction = 0.0271). No other significant interactions were observed (Figure 2).

### Sensitivity analysis

Individuals with zinc supplementation had a reduced risk of dementia [HR (95% CI): 0.79 (0.67–0.92)] and Alzheimer’s disease [0.71 (0.54–0.92)] compared to age- and sex-matched controls (Supplementary Table S2).

## Discussion

In this large population of community-dwelling adults, zinc supplementation, but not other antioxidants, was associated with a reduced risk of dementia and/or Alzheimer’s disease. This inverse association between zinc supplementation and the incidence of dementia/Alzheimer’s disease was further confirmed in an age- and sex-matched analysis. Notably, the association between zinc supplementation and incident dementia was stronger among individuals with cataract.

The consumption of natural antioxidants may reduce oxidative stress, thereby mitigating the risk factors of dementia including hypertension, diabetes, dyslipidemia, and cardiovascular disease (14). However, recent studies have not found significant associations between antioxidant consumption and the risk of cardiometabolic disorders (3, 15). Likely, supplementation with vitamins C or E was not significantly associated with the risk of dementia (7). Several cross-sectional studies have reported a positive association between selenium supplementation and cognition (9, 16, 17). Whilst a recent mendelian randomization analysis found that selenium supplementation was not significantly associated with the risk of Alzheimer’s disease (18). Likely, we did not find a significant association between selenium supplementation and the incidence of dementia or its phenotypes.

We found that zinc supplementation was associated with a lower risk of all-cause dementia and Alzheimer’s disease, independent of demographic information, lifestyle factors, APOE4, cardiometabolic disorders, and depression. A cross-sectional analysis of 591 community-dwelling older adults found that zinc deficiency was associated with a higher prevalence of cognitive decline (19). In another cross-sectional study of 2,450 participants aged 60 years or older from the National Health and Nutrition Examination Survey 2011–2014, zinc intake was non-linearly associated with cognitive function (9). Data from the National Health and Nutrition Examination Survey 2011–2014 also revealed an inverse association between dietary zinc intake and the prevalence of low cognitive performance (16). There are several mechanisms for the potential favorable effects of zinc supplementation on the prevention of dementia/Alzheimer’s disease. Firstly, zinc exhibits antioxidant properties, protecting neurons from oxidative stress and reducing neuroinflammation, thus mitigating the risk of cognitive decline and neurodegenerative diseases (1). Meanwhile, zinc may act as a cofactor for various enzymes and transcription factors involved in synaptic plasticity, which is crucial for learning and memory (20, 21). Thirdly, zinc may play a role in neurotransmitter regulation, which is critical for memory formation and attention (22). However, our findings need to be confirmed in future longitudinal studies.

Although the inverse association between zinc supplementation and incident dementia was significant in individuals with and without cataract, this association was stronger among those with cataract. As an antioxidant, zinc may help to combat oxidative stress in the lens, potentially protecting against the damage to lens proteins and impairing their function thus reducing the risk of developing cataracts (23). Evidence has demonstrated that zinc supplementation may reduce the risk of eye diseases and promote visual function (24, 25). While cataracts have been associated with a higher risk of dementia (26). Therefore, zinc supplementation may reduce oxidative stress, potentially delaying or preventing the risk of dementia directly and the risk caused by cataract simultaneously. This partly explains why the potential beneficial effects of zinc supplementation are stronger among those with cataract.

To our knowledge, this is the first large cohort study to examine the association between antioxidant supplementation and the incidence of dementia. The present study has several limitations. Firstly, although previous research has demonstrated a good agreement between dementia case ascertainment with inpatient data (death register) and primary care records (27), it is possible that some dementia cases might not be captured by inpatient and mortality data. Secondly, the results of our study do not support the establishment of causal relationships because of the observational design. Thirdly, while several cross-sectional studies have explored the potential dose–response relationship of antioxidant supplementation in relation to cognitive impairment (8, 16, 19), there is relatively limited information available about the link between the duration of antioxidant supplementation and cognitive impairment or dementia (9, 16). We could not examine these associations in our study given the lack of relevant data. Fifthly, the age at which antioxidant supplementation started might be significant in the context of dementia development, but we did not investigate this aspect in our study due to the absence of source data. Sixthly, numerous other antioxidants, including vitamin A, beta-carotene, anthocyanins, and lycopene, were not included in our study due to the lack of pertinent data. Finally, most of the participants in our analyses were white Europeans in the United Kingdom thus our findings may not be generalized to whites in developing countries or other ethnic groups.

In conclusion, we found the supplementation of zinc, but not other antioxidants, was significantly associated with the incidence of dementia. Our findings suggest that zinc supplementation may help to mitigate the risk of all-cause dementia and Alzheimer’s disease in middle-aged or older adults, especially among those with cataract. Future research needs to examine the association between the dose and duration of zinc supplementation and the risk of dementia.

## Data availability statement

The original contributions presented in the study are included in the article/Supplementary material, further inquiries can be directed to the corresponding authors.

## Ethics statement

The studies involving humans were approved by The National Information Governance Board for Health and Social Care and the NHS North West Multicenter Research Ethics Committee. The studies were conducted in accordance with the local legislation and institutional requirements. The participants provided their written informed consent to participate in this study.

## Author contributions

XS: Conceptualization, Formal Analysis, Methodology, Software, Visualization, Writing – review & editing. JL: Formal Analysis, Visualization, Writing – original draft. XZ: Visualization, Writing – original draft, Writing – review & editing. YH: Visualization, Writing – original draft, Writing – review & editing. ZZ: Data curation, Visualization, Writing – original draft, Writing – review & editing. ST: Visualization, Writing – original draft, Writing – review & editing. WW: Data curation, Project administration, Writing – original draft, Writing – review & editing. ZG: Formal Analysis, Methodology, Writing – original draft, Writing – review & editing. HY: Funding acquisition, Supervision, Writing – original draft. MH: Conceptualization, Funding acquisition, Resources, Supervision, Writing – original draft, Writing – review & editing.
