# Supplementary material for: Association of antioxidants use with the risk of dementia among community-dwelling adults in the United Kingdom biobank
Source: Front Nutr. 2024 Jan 4;10:1270179. doi: 10.3389/fnut.2023.1270179 (PMC10794302; doi:10.3389/fnut.2023.1270179)
Supplement: Supplementary file 1 [file Data_Sheet_1.docx]

Table S1. Codes for international classification disease and self-reported fields for dementia

Table S2. Zinc supplementation and the risk for incident dementia in zinc users and age- and sex-matched controls

**Table S1. Codes for international classification disease and self-reported fields for dementia**

| **Type of dementia** | **ICD-9*** | **ICD-10** | **Self-reported fields** |
| --- | --- | --- | --- |
| All-cause dementia | 331·0, 290·4, 331·1, 290·2, 290·3, 291·2, 294·1, 331·2, 331·5 | F00, F00·0, F00·1, F00·2, F00·9, G30, G30·0, G30·1, G30·8, G30·9, F01, F01·0, F01·1, F01·2, F01·3, F01·8, F01·9, I67·3, F02·0, G31·0, A81·0, F02, F02·1, F02·2, F02·3, F02·4, F02·8, F03, F05·1, F10·6, G31·1, G31·8 | 1263 |
| Alzheimer’s disease | 331·0 | F00, F00·0, F00·1, F00·2, F00·9, G30, G30·0, G30·1, G30·8, G30·9 |  |
| Vascular dementia | 290·4 | F01, F01·0, F01·1, F01·2, F01·3, F01·8, F01·9 |  |

**Primary and secondary diagnosis from hospital records were used to identify dementia cases.*

**Table S2. Zinc supplementation and the risk for incident dementia in zinc users and age- and sex-matched controls**

|  | Zinc use | |  | P-value |
| --- | --- | --- | --- | --- |
|  | No | Yes |  |  |
| **Dementia** |  |  |  |  |
| Events | 902 | 248 |  |  |
| Person-years | 729012 | 243193 |  |  |
| HR (95% CI), Model 1 | Reference | 0.87 (0.75-1.00) |  | 0.0516 |
| HR (95% CI), Model 2 | Reference | 0.79 (0.67-0.93) |  | 0.0037 |
| **Alzheimer's disease** | |  |  |  |
| Events | 357 | 87 |  |  |
| Person-years | 729012 | 243193 |  |  |
| HR (95% CI), Model 1 | Reference | 0.76 (0.60-0.96) |  | 0.0220 |
| HR (95% CI), Model 2 | Reference | 0.61 (0.47-0.80) |  | 0.0003 |
| **Vascular dementia** | |  |  |  |
| Events | 163 | 44 |  |  |
| Person-years | 729012 | 243193 |  |  |
| HR (95% CI), Model 1 | Reference | 0.88 (0.63-1.23) |  | 0.45 |
| HR (95% CI), Model 2 | Reference | 0.85 (0.58-1.24) |  | 0.39 |

Hazard ratio (95% CI) for incident dementia associated with antioxidants supplementation was estimated using Cox proportional regression models. Model 1 was adjusted for APOE4, ethnicity, education, income, diet score, smoking, alcohol consumption, sleep duration, physical activity, BMI, depression, dyslipidemia, hypertension, diabetes, and stroke at baseline; Model 2 was adjusted for Model 1 plus calcium, glucosamine, and fish oil supplements.
